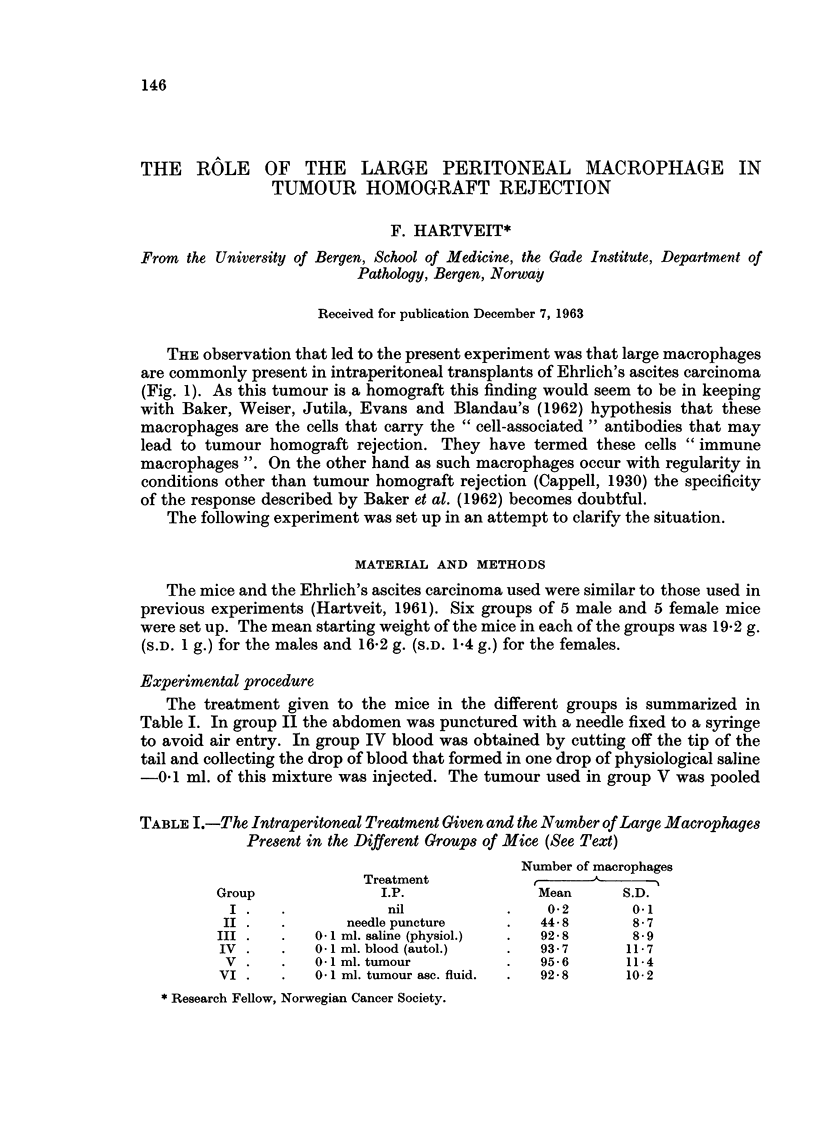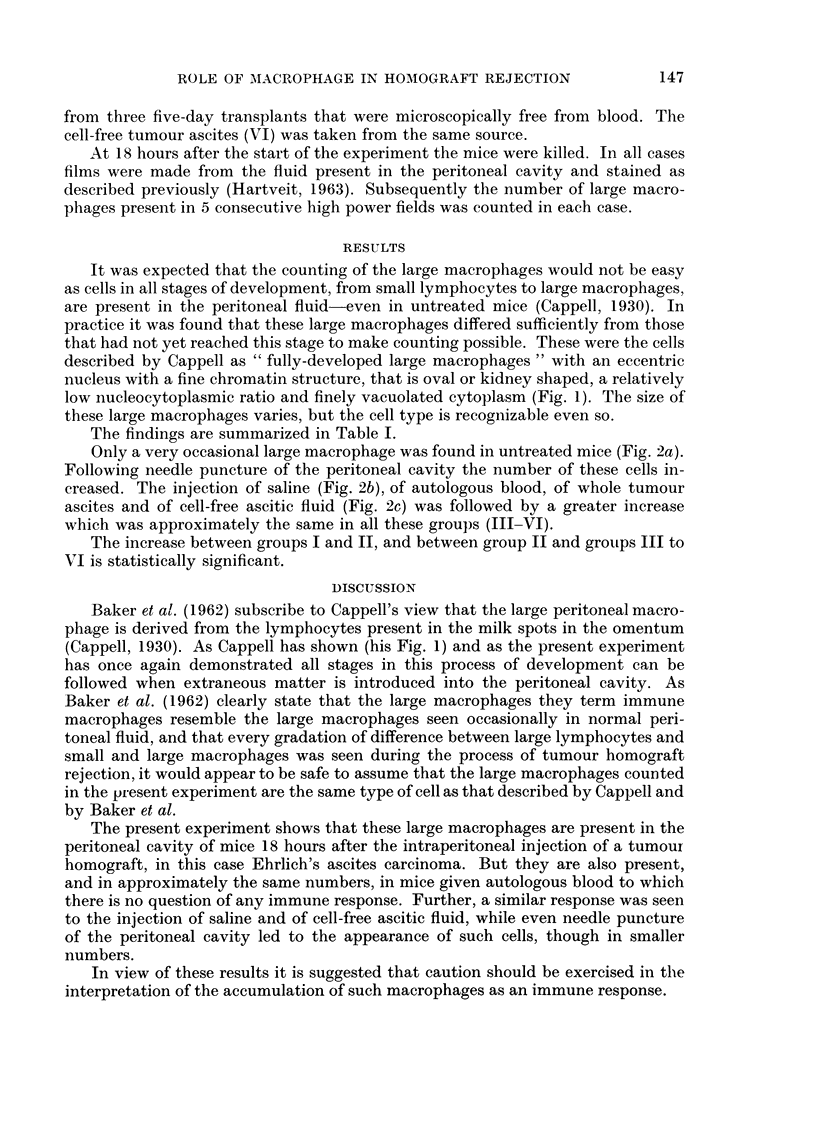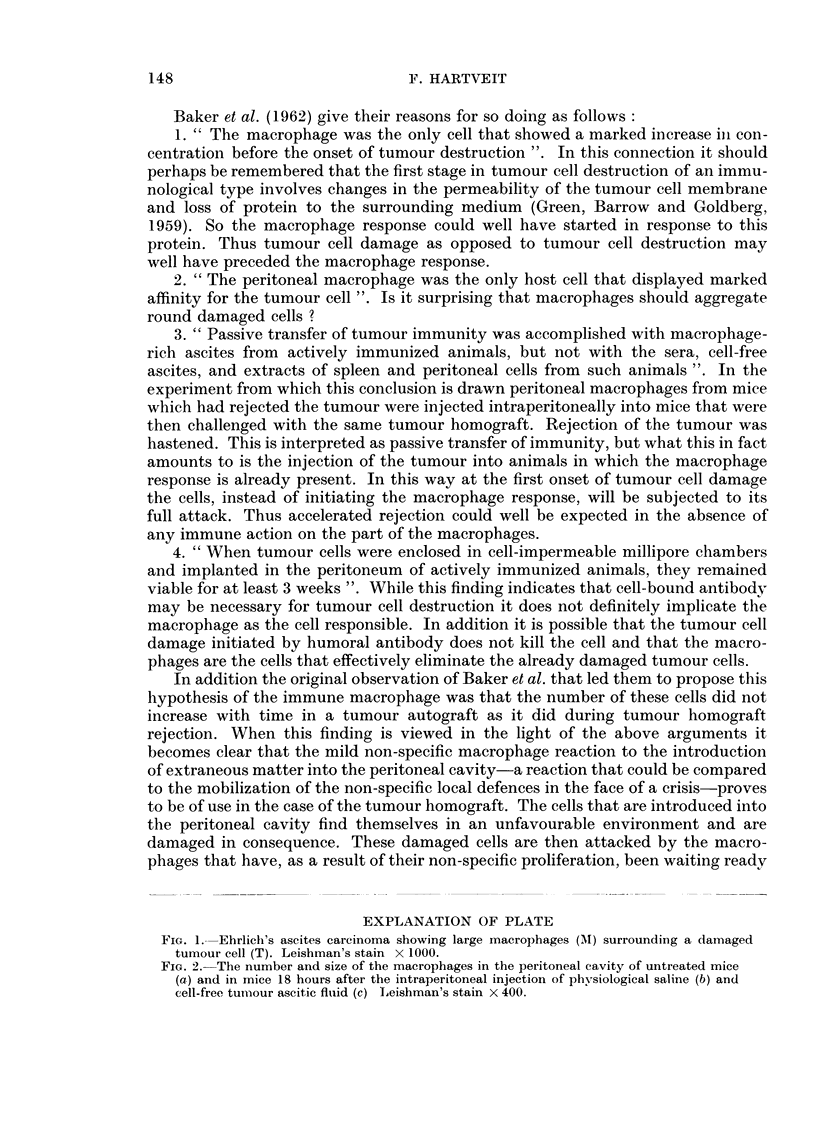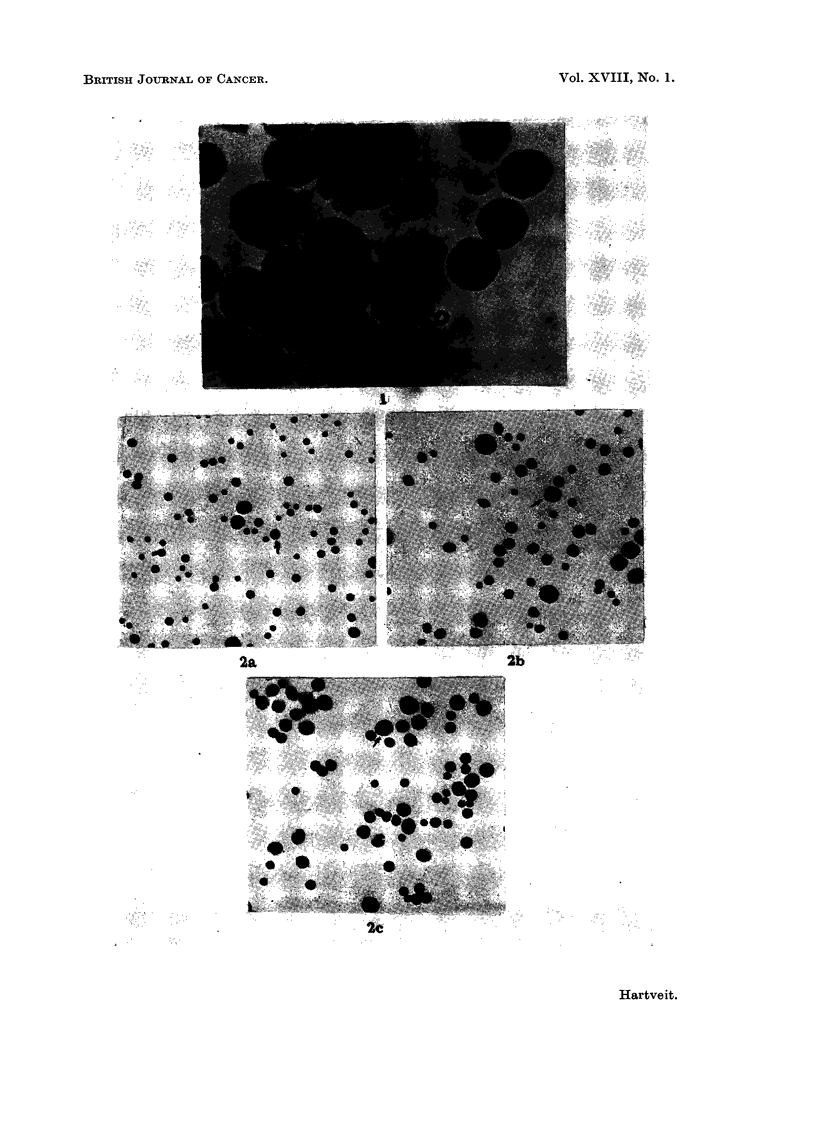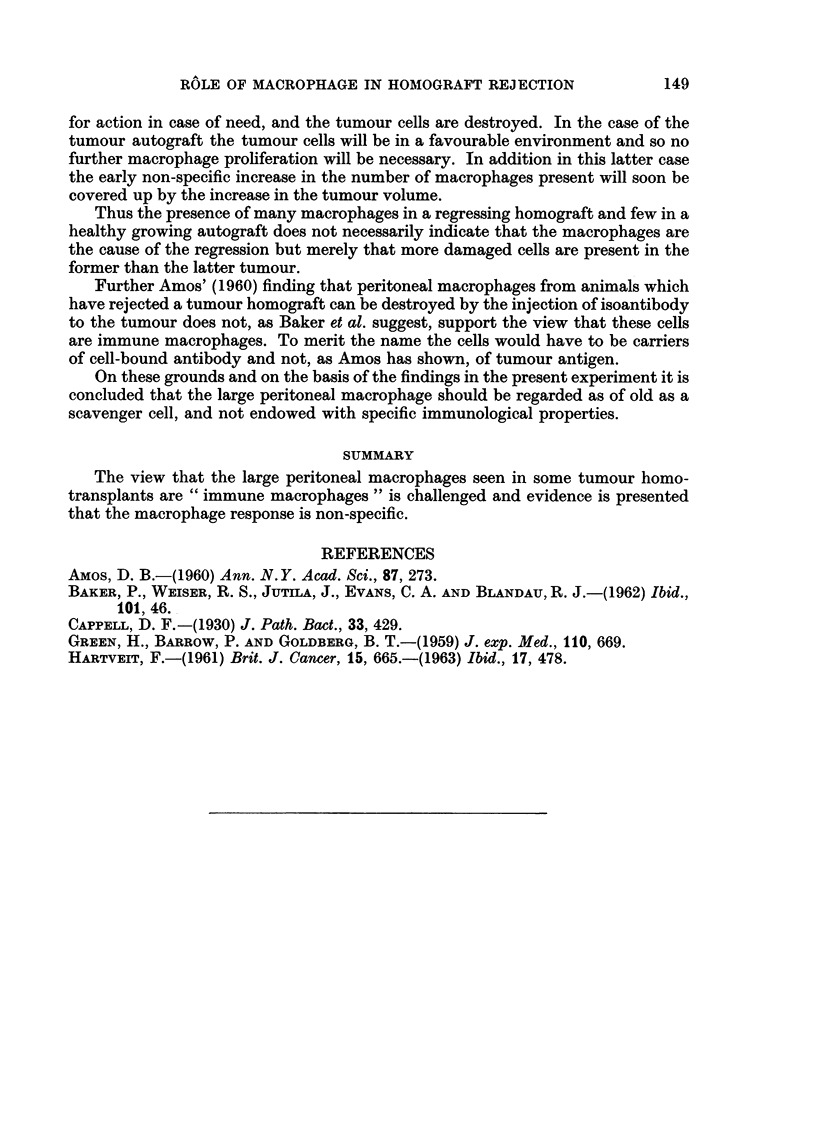# The Rôle of the Large Peritoneal Macrophage in Tumour Homograft Rejection

**DOI:** 10.1038/bjc.1964.16

**Published:** 1964-03

**Authors:** F. Hartveit

## Abstract

**Images:**


### THE RÔLE OF THE LARGE PERITONEAL MACROPHAGE IN TUMOUR HOMOGRAFT REJECTION

F. HARTVEIT*

*From the University of Bergen, School of Medicine, the Gade Institute, Department of Pathology, Bergen, Norway*

Received for publication December 7, 1963

THE observation that led to the present experiment was that large macrophages are commonly present in intraperitoneal transplants of Ehrlich's ascites carcinoma (Fig. 1). As this tumour is a homograft this finding would seem to be in keeping with Baker, Weiser, Jutila, Evans and Blandau's (1962) hypothesis that these macrophages are the cells that carry the " cell-associated " antibodies that may lead to tumour homograft rejection. They have termed these cells " immune macrophages ". On the other hand as such macrophages occur with regularity in conditions other than tumour homograft rejection (Cappell, 1930) the specificity of the response described by Baker *et al.* (1962) becomes doubtful.

The following experiment was set up in an attempt to clarify the situation.

### MATERIAL AND METHODS

The mice and the Ehrlich's ascites carcinoma used were similar to those used in previous experiments (Hartveit, 1961). Six groups of 5 male and 5 female mice were set up. The mean starting weight of the mice in each of the groups was 19·2 g. (S.D. 1 g.) for the males and 16·2 g. (S.D. 1·4 g.) for the females.

### *Experimental procedure*

The treatment given to the mice in the different groups is summarized in Table I. In group II the abdomen was punctured with a needle fixed to a syringe to avoid air entry. In group IV blood was obtained by cutting off the tip of the tail and collecting the drop of blood that formed in one drop of physiological saline —0·1 ml. of this mixture was injected. The tumour used in group V was pooled

TABLE I.—*The Intraperitoneal Treatment Given and the Number of Large Macrophages Present in the Different Groups of Mice (See Text)*

| Group | Treatment I.P. | Number of macrophages Mean | S.D. |
|---|---|---|---|
| I | nil | 0·2 | 0·1 |
| II | needle puncture | 44·8 | 8·7 |
| III | 0·1 ml. saline (physiol.) | 92·8 | 8·9 |
| IV | 0·1 ml. blood (autol.) | 93·7 | 11·7 |
| V | 0·1 ml. tumour | 95·6 | 11·4 |
| VI | 0·1 ml. tumour asc. fluid. | 92·8 | 10·2 |

* Research Fellow, Norwegian Cancer Society.

from three five-day transplants that were microscopically free from blood. The cell-free tumour ascites (VI) was taken from the same source.

At 18 hours after the start of the experiment the mice were killed. In all cases films were made from the fluid present in the peritoneal cavity and stained as described previously (Hartveit, 1963). Subsequently the number of large macrophages present in 5 consecutive high power fields was counted in each case.

### RESULTS

It was expected that the counting of the large macrophages would not be easy as cells in all stages of development, from small lymphocytes to large macrophages, are present in the peritoneal fluid—even in untreated mice (Cappell, 1930). In practice it was found that these large macrophages differed sufficiently from those that had not yet reached this stage to make counting possible. These were the cells described by Cappell as " fully-developed large macrophages " with an eccentric nucleus with a fine chromatin structure, that is oval or kidney shaped, a relatively low nucleocytoplasmic ratio and finely vacuolated cytoplasm (Fig. 1). The size of these large macrophages varies, but the cell type is recognizable even so.

The findings are summarized in Table I.

Only a very occasional large macrophage was found in untreated mice (Fig. 2*a*). Following needle puncture of the peritoneal cavity the number of these cells increased. The injection of saline (Fig. 2*b*), of autologous blood, of whole tumour ascites and of cell-free ascitic fluid (Fig. 2*c*) was followed by a greater increase which was approximately the same in all these groups (III–VI).

The increase between groups I and II, and between group II and groups III to VI is statistically significant.

### DISCUSSION

Baker *et al.* (1962) subscribe to Cappell's view that the large peritoneal macrophage is derived from the lymphocytes present in the milk spots in the omentum (Cappell, 1930). As Cappell has shown (his Fig. 1) and as the present experiment has once again demonstrated all stages in this process of development can be followed when extraneous matter is introduced into the peritoneal cavity. As Baker *et al.* (1962) clearly state that the large macrophages they term immune macrophages resemble the large macrophages seen occasionally in normal peritoneal fluid, and that every gradation of difference between large lymphocytes and small and large macrophages was seen during the process of tumour homograft rejection, it would appear to be safe to assume that the large macrophages counted in the present experiment are the same type of cell as that described by Cappell and by Baker *et al.*

The present experiment shows that these large macrophages are present in the peritoneal cavity of mice 18 hours after the intraperitoneal injection of a tumour homograft, in this case Ehrlich's ascites carcinoma. But they are also present, and in approximately the same numbers, in mice given autologous blood to which there is no question of any immune response. Further, a similar response was seen to the injection of saline and of cell-free ascitic fluid, while even needle puncture of the peritoneal cavity led to the appearance of such cells, though in smaller numbers.

In view of these results it is suggested that caution should be exercised in the interpretation of the accumulation of such macrophages as an immune response.

Baker *et al.* (1962) give their reasons for so doing as follows :

1. " The macrophage was the only cell that showed a marked increase in concentration before the onset of tumour destruction ". In this connection it should perhaps be remembered that the first stage in tumour cell destruction of an immunological type involves changes in the permeability of the tumour cell membrane and loss of protein to the surrounding medium (Green, Barrow and Goldberg, 1959). So the macrophage response could well have started in response to this protein. Thus tumour cell damage as opposed to tumour cell destruction may well have preceded the macrophage response.

2. " The peritoneal macrophage was the only host cell that displayed marked affinity for the tumour cell ". Is it surprising that macrophages should aggregate round damaged cells ?

3. " Passive transfer of tumour immunity was accomplished with macrophage-rich ascites from actively immunized animals, but not with the sera, cell-free ascites, and extracts of spleen and peritoneal cells from such animals ". In the experiment from which this conclusion is drawn peritoneal macrophages from mice which had rejected the tumour were injected intraperitoneally into mice that were then challenged with the same tumour homograft. Rejection of the tumour was hastened. This is interpreted as passive transfer of immunity, but what this in fact amounts to is the injection of the tumour into animals in which the macrophage response is already present. In this way at the first onset of tumour cell damage the cells, instead of initiating the macrophage response, will be subjected to its full attack. Thus accelerated rejection could well be expected in the absence of any immune action on the part of the macrophages.

4. " When tumour cells were enclosed in cell-impermeable millipore chambers and implanted in the peritoneum of actively immunized animals, they remained viable for at least 3 weeks ". While this finding indicates that cell-bound antibody may be necessary for tumour cell destruction it does not definitely implicate the macrophage as the cell responsible. In addition it is possible that the tumour cell damage initiated by humoral antibody does not kill the cell and that the macrophages are the cells that effectively eliminate the already damaged tumour cells.

In addition the original observation of Baker *et al.* that led them to propose this hypothesis of the immune macrophage was that the number of these cells did not increase with time in a tumour autograft as it did during tumour homograft rejection. When this finding is viewed in the light of the above arguments it becomes clear that the mild non-specific macrophage reaction to the introduction of extraneous matter into the peritoneal cavity—a reaction that could be compared to the mobilization of the non-specific local defences in the face of a crisis—proves to be of use in the case of the tumour homograft. The cells that are introduced into the peritoneal cavity find themselves in an unfavourable environment and are damaged in consequence. These damaged cells are then attacked by the macrophages that have, as a result of their non-specific proliferation, been waiting ready

---

EXPLANATION OF PLATE

FIG. 1.—Ehrlich's ascites carcinoma showing large macrophages (M) surrounding a damaged tumour cell (T). Leishman's stain $\times$ 1000.

FIG. 2.—The number and size of the macrophages in the peritoneal cavity of untreated mice (*a*) and in mice 18 hours after the intraperitoneal injection of physiological saline (*b*) and cell-free tumour ascitic fluid (*c*) Leishman's stain $\times$ 400.

1

2a

2b

2c

Hartveit.

for action in case of need, and the tumour cells are destroyed. In the case of the tumour autograft the tumour cells will be in a favourable environment and so no further macrophage proliferation will be necessary. In addition in this latter case the early non-specific increase in the number of macrophages present will soon be covered up by the increase in the tumour volume.

Thus the presence of many macrophages in a regressing homograft and few in a healthy growing autograft does not necessarily indicate that the macrophages are the cause of the regression but merely that more damaged cells are present in the former than the latter tumour.

Further Amos' (1960) finding that peritoneal macrophages from animals which have rejected a tumour homograft can be destroyed by the injection of isoantibody to the tumour does not, as Baker *et al.* suggest, support the view that these cells are immune macrophages. To merit the name the cells would have to be carriers of cell-bound antibody and not, as Amos has shown, of tumour antigen.

On these grounds and on the basis of the findings in the present experiment it is concluded that the large peritoneal macrophage should be regarded as of old as a scavenger cell, and not endowed with specific immunological properties.

SUMMARY

The view that the large peritoneal macrophages seen in some tumour homotransplants are " immune macrophages " is challenged and evidence is presented that the macrophage response is non-specific.

### REFERENCES

Amos, D. B.—(1960) *Ann. N.Y. Acad. Sci.*, **87**, 273.
Baker, P., Weiser, R. S., Jutila, J., Evans, C. A. and Blandau, R. J.—(1962) *Ibid.*, **101**, 46.
Cappell, D. F.—(1930) *J. Path. Bact.*, **33**, 429.
Green, H., Barrow, P. and Goldberg, B. T.—(1959) *J. exp. Med.*, **110**, 669.
Hartveit, F.—(1961) *Brit. J. Cancer*, **15**, 665.—(1963) *Ibid.*, **17**, 478.